# Effects of an Immunomodulatory Supplement and Evaporative Cooling on Immune Status, Mammary Gland Microstructure, and Gene Expression of Cows Exposed to Heat Stress During the Dry Period

**DOI:** 10.3390/ani15213113

**Published:** 2025-10-27

**Authors:** Thiago F. Fabris, Jimena Laporta, Fabiana N. Corra, Yazielis M. Torres, David J. Kirk, James D. Chapman, Geoffrey E. Dahl

**Affiliations:** 1 Department of Animal Sciences, University of Florida, P.O. Box 110910, Gainesville, FL 32611, USA; 2 Phibro Animal Health Corporation, Teaneck, NJ 07666, USA

**Keywords:** heat stress, dry period, mammary development, immunity

## Abstract

**Simple Summary:**

Heat stress during the dry period reduces immune function, mammary growth, and subsequent milk yield. Mammary involution and regrowth are linked with immune function. Therefore, supplementation with an immunomodulator during involution may reverse some negative effects of heat stress on mammary development. Our previous study demonstrated that supplementing OmniGen-AF^®^ (OMN) before, during, and after the dry period to heat-stressed dairy cows lowers respiration rate and rectal temperature and improves milk yield in the subsequent lactation. In this study, OMN supplementation during the dry period produced effects on mammary gene expression, regrowth, and hormonal shifts that were comparable to those observed with the cooling of heat-stressed dairy cows.

**Abstract:**

Nutritional and cooling strategies to abate the negative effects of heat stress during the dry period have been used to improve the performance of dairy cattle. The objective of this study was to evaluate the effects of feeding an immunomodulatory supplement (OmniGen-AF^®^, OMN) before, during, and after exposure to either heat stress or active cooling during the dry period on immune function and mammary development in dairy cows. During late lactation (at least 60 d before dry off), cows were provided with evaporative cooling systems (shade, fans, and soakers) and assigned to two groups: placebo (56 g/d of AB20^®^ top-dressed; CON) or OmniGen-AF^®^ (OMN, 56 g/d top-dressed). Cows were dried off ~46 d before the expected calving date and further split into evaporative cooling (shade, fans, and soakers; CL) or heat stress (only shade; HT) pens. Thus, after dry off, there were four treatment groups: heat stress with placebo (HT, n = 17), HT with OMN supplementation (HT + OMN, n = 19), CL with placebo (CL, n = 16), and CL with OMN supplementation (CL + OMN, n = 11). From a subset of cows (n = 6–8 per group), four blood samples were collected during the dry period (−43, −39, −32, and −21 d relative to calving) to evaluate neutrophil function and blood hematology. In addition, mammary biopsies (4–6 cows/treatment) were collected at −43, −39, −32, and −21 d relative to calving to evaluate mammary gland gene expression and histology, i.e., Tdt dUTP nick-end labeling (TUNEL) and Ki67. Genes related to autophagy, apoptosis, and cell proliferation were analyzed by qRT-PCR. Relative to CL, HT downregulated the expression of beclin-2 (*BECN2*) but upregulated the expression of beclin-1 (*BECN1*) on days −43 and −39 relative to calving, respectively. Also, relative to CL, HT upregulated the expression of *BAX* and *FAS* on day −39 relative to calving. These differences in gene expression were followed by HT cows having a lower total cell apoptosis rate during involution relative to CL cows. Further to these effects, HT leads to a lower alveoli number relative to CL cows. As in the CL treatment, OMN cows have a higher total cell apoptosis rate and alveoli number relative to CON cows. In addition, OMN cows have higher total cell proliferation relative to CON. Prolactin (PRL) and cortisol concentrations were evaluated during the dry period at days −45, −26, −3, and −1 relative to calving. Relative to CL, HT cows had higher PRL at day −45 but lower PRL on day −1 relative to calving, and a similar trend was observed for cortisol concentrations. In summary, HT impacts mammary gland gene expression, compromises mammary involution, reduces alveoli number, and alters hormone dynamics throughout the dry period. Following the same trends as the CL treatment, OMN increases mammary gland turnover by having a higher cell apoptosis and cell proliferation rate and lower connective tissue relative to CON cows.

## 1. Introduction

The dry period, defined as the non-lactating period of dairy cows, is important for the full recovery of mammary gland tissue to support maximal milk yield in the next lactation. Dairy cows exposed to heat stress during the dry period have impaired milk yield in the next lactation [1,2], which negatively impacts profitability. Mammary cell number and secretory capacity per cell are related to milk yield. Heat stress during the dry period compromises mammary gland growth [3,4] and consequently reduces the capacity for milk yield in the subsequent lactation [5]. Thus, mammary gland development during the dry period may be important for increasing milk yield in the next lactation [6].

After dry off at the cessation of milk removal, the mammary gland undergoes rapid changes known as involution. Mammary gland involution is a cellular remodeling process of the gland that is mediated by programmed cell death (i.e., apoptosis; [6,7]). The dry period has been divided into two phases, the early dry period (involution) and the late dry period (cell redevelopment; [3,6,8]). In the early dry period, mammary gland involution is driven by autophagy and apoptosis [9,10]. Apoptosis and autophagy are mechanisms responsible for the destruction and recycling of cells, respectively. Both processes are linked to immune activity, and thus, immune competency may alter their progression and accelerate cell turnover during the dry period, and ultimately affect mammary development.

Determining the relationship between heat stress during the dry period and the reduced immune status of cows may be important to understanding the impact of immune system components and the process of involution in the mammary gland early in the dry period and subsequent mammary cell proliferation prior to parturition. Strategies that improve the immune status of dairy cows under heat stress conditions may result in higher cell turnover and are a useful strategy to reduce some of the negative effects of heat stress. OmniGen-AF^®^ is a feed product (Phibro Animal Health Corporation, Teaneck, NJ, USA) with immunomodulatory capacity that has been shown to ameliorate the negative effects of heat stress during the dry period and improve subsequent lactation performance [2]. In addition, Brandão et al. [11] demonstrated that OMN supplementation increased polymorphonuclear (PMN) cells in the endometrium, and increased TNFα and haptoglobin in response to lipopolysaccharide (LPS) administration, which was indicated as enhanced immunocompetence of dairy cows during the transition period. Thus, the objective of the present study was to evaluate the effect of feeding OmniGen-AF^®^ during late lactation, and during the dry period to cows exposed to either heat stress or evaporative cooling when dry, on immune status and subsequent mammary gland development. Our hypothesis was that cooling and OmniGen-AF^®^ would elicit similar effects on mammary gland development and gene expression in cows exposed to heat stress in late gestation.

## 2. Materials and Methods

### 2.1. Treatments, Experimental Design, and Animals

The experiment was conducted for one summer (April to October 2015) at the University of Florida Dairy Unit (Hague, FL, USA). Data presented here were collected from animals enrolled in a larger study, and the production-related responses have been reported previously [2]. All treatments and procedures were approved by the University of Florida IACUC. A 2 × 2 factorial design was used to evaluate the effects of supplemental feeding of a placebo (56 g/d of AB20^®^ Phibro Animal Health Corporation; CON) or OmniGen-AF^®^ (56 g/d of OMN) on the immune status of dairy cattle experiencing heat stress (HT) or cooling (CL) during the dry period. The feed additive OmniGen-AF^®^ contains a mixture of silicon dioxide, calcium aluminosilicate, sodium aluminosilicate, Brewer’s dehydrated yeast, mineral oil, calcium carbonate, rice hulls, niacin supplement, biotin, d-calcium pantothenate, vitamin B-12 supplement, choline chloride, thiamine mononitrate, pyridoxine hydrochloride, riboflavin-5-phosphate, and folic acid, but the full formulation is proprietary. During late lactation (at least 60 d before dry off), cows were randomly assigned to dietary treatments (OMN or CON) based on mature equivalent milk yield in the previous lactation. Cows were then dried off ~46 d before expected calving, and CON and OMN treatments continued when cows were exposed to temperature (i.e., HT or CL) treatments when dry. Temperature and dietary treatment groups were as follows: heat stress, access only to a shaded free-stall barn (HT, n = 17, 56 g/d of AB20^®^, 204.7 ± 22.1 d on supplementation), HT with OMN (HT + OMN, n = 19, 56 g/d of OMN, 213 ± 25.7 d on supplementation), cooling with access to a shaded free-stall barn with fans and soakers (CL, n = 16, 56 g/d of AB20^®^, 199.8 ± 24.2 d on supplementation), and CL with OMN (CL + OMN, n = 11, 56 g/d of OMN, 211.9 ± 21.1 d on supplementation).

Cows were housed in a sand-bedded free-stall barn during lactation and the dry period. The THI was calculated based on the equation reported by Dikmen et al. [12], THI = (1.8 × T + 32) − [(0.55 − 0.0055 × RH) × (1.8 × T − 26)], where T = air temperature (°C) and RH = relative humidity (%). During the dry period, the pens for CL and CL + OMN treatments were equipped with shade and active cooling, including soakers (Rain Bird Manufacturing, Glendale, CA, USA) and fans (J&D Manufacturing, Eau Claire, WI, USA), whereas the pens for HT and HT + OMN treatments only had shade. When the ambient temperature exceeded 21.1 °C, fans automatically turned on, and the soakers were activated for 1.5 min at 5 min intervals. The photoperiod (14 h light/10 h dark) of the barn for dry cows on all treatments was similar and controlled using metal halide lights. The lights provided approximately 250 lux intensity at the eye level of cows and were kept on from 0600 to 2000 h. After calving, all cows were housed in the same sand-bedded free-stall barn with soakers and fans for cooling, and all cows had access to cooling. The air temperature and relative humidity of each pen in the barn for dry cows were recorded every 15 min by Hobo Pro series Temp probes (Onset Computer Corp., Pocasset, MA, USA). All cows were fed a common close-up total mixed ration TMR (diet ingredients on a percentage dry matter basis consisted of corn silage (50%), Bermuda grass hay (17%), Brewer’s grains (20%), soybean hulls (3.4%), mineral premix (4.0%), SoyChlor (5.0%), and mycotoxin binder (0.6%), as reported in [2]) during the entire dry period and daily DMI of individual cows was measured from dry off to calving. Rectal temperature (RT) was measured twice daily (0730 and 1430), and RR was counted thrice weekly (1400 h, Monday–Wednesday–Friday) for all cows during the dry period and was described by Fabris et al. [2].

### 2.2. Mammary Gland Biopsy and Histology

Mammary gland biopsies were collected during late lactation 3 days before dry off (~46 d before expected calving) and on days −43, −39, −32, and −21 relative to calving from a subset of cows (n = 4–6 cows per treatment). Mammary gland collection was performed as described by Tao et al. [3]. After collection, mammary gland samples were divided for immunohistochemistry and gene expression analysis. For staining and immunohistochemistry, one portion of mammary gland tissue was placed in cassettes and fixed overnight at 4 °C in 4% paraformaldehyde and then submitted to a series of ethanol rinses (25%, 50%, 70%, and 100%) and transferred to 70% ethanol and kept at 4 °C until all samples were collected and paraffin embedded following the procedures of the University of Florida Molecular Pathology Core. Following embedding, tissues were then sectioned at 5 μm onto slides coated with poly-L-lysine and stained with hematoxylin and eosin (H&E), Masson’s trichrome, and immunohistochemistry (Ki67) by the Molecular Pathology Core to visualize morphology, connective tissue area, and proliferating cells. Also, terminal deoxynucleotidyl transferase dUTP nick-end labeling (TUNEL) was performed, using immunohistochemistry as previously reported by Tao et al. [3]. For gene expression analysis, samples were cut and stored in RNA Later (Qiagen, Germantown, MD, USA) for 24 h and then kept at −80 °C until RNA extraction. Mammary gland tissue (50–70 mg) was used for total RNA, and it was extracted using the RNeasy Plus Universal Mini Kit (Qiagen) according to the manufacturer’s instructions. A quality check was performed using a NanoDrop^®^1000 Spectrophotometer (Wilmington, DE, USA), and samples were evaluated by optical density measurement (A260/A280 ratio above 1.8 for all samples). For cDNA synthesis, the same amount of extracted RNA was used, and a cDNA reverse transcription kit was used (iScript Bio-Rad, Cat. No. 170-8441; Hercules, CA, USA). Then, quantitative real-time PCR (qRT-PCR) was conducted using the CFX96 Touch Real-Time PCR Detection System (Bio-Rad) for the genes represented in Table 1.

### 2.3. Cortisol and Prolactin Analysis

For prolactin, blood samples were collected on the day of dry off, when temperature treatments (i.e., CL vs. HT) were applied (~−46 d before calving), on day 1 dry (~−45 d before calving), the mid-dry period (~−26 d relative to calving), and as parturition approached (−3 d and −1 d before calving; n = 13 cows/treatment). Blood samples were collected from coccygeal vessels into 10 mL EDTA vacutainers (BD Vacutainer^®^ spray-coated K2EDTA Tubes; Becton-Dickinson, Franklin Lakes, NJ, USA). Samples were then placed on ice and centrifuged at 2619× *g* at 4 °C for 20 min within 1 h after collection, and plasma was collected. Plasma samples were then stored at −20 °C until samples were analyzed for prolactin and cortisol concentrations. Cortisol samples were analyzed using a chemiluminescent enzyme immunoassay (Immulite 1000; Siemens Medical Solutions Diagnostics, Los Angeles, CA, USA). Prolactin was measured by using an Enzyme-Linked Immunosorbent assay kit (Cloud-Clone Corp., Houston, TX, USA; catalog number, SEA846Bo) according to the manufacturer’s procedure.

### 2.4. Statistical Analysis

Data were analyzed as a completely randomized design using the PROC MIXED or GLIMMIX procedure of SAS version 9.4 (SAS Institute Inc., Cary, NC, USA), and least squares means ± SEM are reported and were compared using PDIFF. All data were tested for normality and adjusted for multiple comparisons using Tukey’s adjustment. The statistical model included the fixed effect of temperature treatment (HT and CL), dietary treatment (CON and OMN), time, and time by treatment interaction with cow (treatment) as a random effect. Also, for mammary cell proliferation and apoptotic rate analysis, the value at 3 d before dry off was used as the baseline and included in the model as a covariate. Differences of 0.05 ≤ *p* ≤ 0.10 were considered as a tendency, and differences of *p* ≤ 0.05 were considered statistically significant.

## 3. Results

### 3.1. Hematology

General immune status, as determined by hematological profiles in blood, is presented in Table 2. Heat stress reduced hematocrit relative to cooling (*p* ≤ 0.02), and OMN feeding did not alter that response. However, hemoglobin was lower with OMN feeding relative to cows that did not receive OMN (*p* ≤ 0.01). The interaction of heat stress and OMN tended to increase platelet counts, but cooling and OMN reduced platelet counts compared with no OMN treatment (*p* ≤ 0.07). In general, immune status was not affected by either temperature or dietary treatment, although significant effects of day was observed as cows moved through the dry period into lactation.

### 3.2. Mammary Gland Cell Turnover and Microstructure

**Figure 1:**
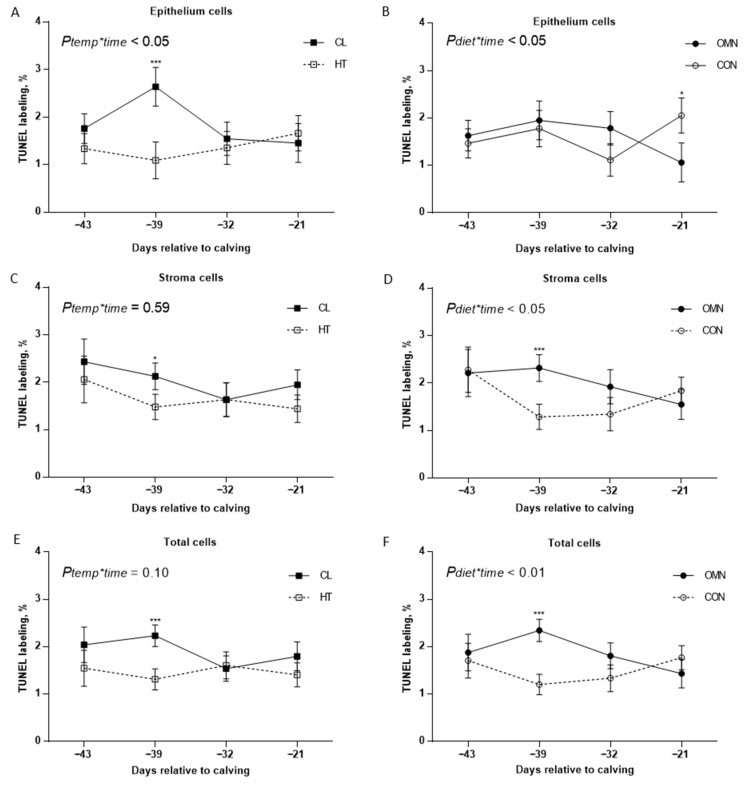
Immunohistochemistry of apoptotic mammary cells in Holstein dairy cows supplemented with OmniGen-AF^®^ and exposed to heat stress in the dry period. Multiparous cows were exposed to temperature treatment (HT and CL, n = 5–6/trt) or dietary treatment (CON and OMN, n = 4–6/trt) during the dry period (~46 d before expected calving). Mammary biopsies were collected at days −43, −39, −32, −21 relative to calving. For apoptosis analysis, the Tdt dUTP nick-end labeling (TUNEL) assay was performed, and mammary gland tissue was visualized at 40×. A proportion of proliferating cells in MEC, stromal, and total mammary cell apoptosis are represented on (**A**–**F**), for temperature and dietary treatment (right and left panel, respectively). Data are presented as LSM ± SEM. * *p* ≤ 0.10; *** *p* ≤ 0.01.

*Terminal deoxynucleotidyl transferase dUTP nick-end labeling (TUNEL).* Epithelium apoptosis analysis showed temperature treatment and time interaction (HT vs. CL, *p* ≤ 0.05) and dietary supplement treatment and time interaction (CON vs. OMN, *p* ≤ 0.05), with limited interactions among temperature, supplement, and time. Compared with CL, HT cows had lower epithelial apoptosis on day −39 relative to calving (*p* ≤ 0.01, Figure 1). Relative to CON, OMN cows tended to have lower epithelial cell apoptosis on day −21 (*p* = 0.08, Figure 1). Compared with CL + OMN; HT + CON and HT + OMN cows had lower epithelial apoptosis on days −39 and −21, respectively (*p* ≤ 0.05), but there was no interaction among temperature, supplement, and time effect (*p* = 0.50). Compared with CL, HT cows tended to have lower stromal apoptosis on day −39 relative to calving (*p* ≤ 0.10, Figure 1). Stromal apoptosis analysis showed a supplement and time effect interaction (*p* ≤ 0.05). Compared with CON, OMN cows had higher stromal apoptosis on day −39 (*p* ≤ 0.01, Figure 1). Also, CL + OMN cows had greater stromal apoptosis relative to HT + CON and CL + CON (*p* ≤ 0.01 and *p* ≤ 0.05), but there was no interaction among temperature, time, and supplement effect (*p* = 0.59). Total mammary cell apoptosis showed an impact of the fixed effect temperature (HT vs. CL, *p* ≤ 0.10), where compared with CL, HT cows had a lower apoptotic percentage. This effect is more accentuated on day −39, where CL cows had higher apoptotic percentage relative to HT (*p* ≤ 0.01, Figure 1). Also, there was an interaction between OMN and time (*p* ≤ 0.01), where OMN cows had a higher apoptotic percentage on day −39 relative to CON cows (*p* ≤ 0.01, Figure 1). Also, CL + CON (1.57 ± 0.21%), HT + CON (1.44 ± 0.23%), and HT + OMN (1.50 ± 0.22%) cows had a lower apoptotic percentage relative to CL + OMN (2.24 ± 0.25%, *p* ≤ 0.05, respectively), but there was no significant interaction among temperature, dietary, and time effects (*p* = 0.25).

**Figure 2:**
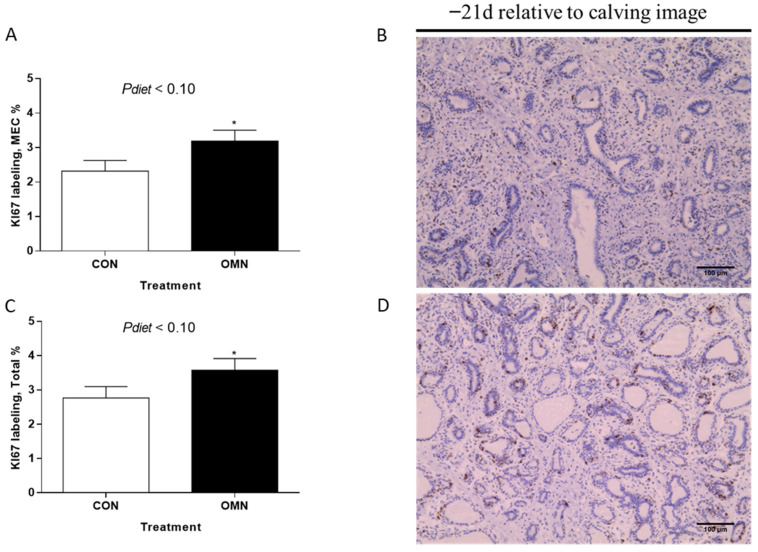
Immunohistochemistry of proliferating mammary cells in Holstein dairy cows supplemented with OmniGen-AF^®^ and exposed to heat stress in the dry period. Multiparous cows were exposed to dietary treatment (CON and OMN, n = 4–6/trt) during the dry period (~46 d before expected calving). Mammary biopsies were collected at days −43, −39, −32, and −21 d relative to calving. For cell proliferation analysis, the Ki67 assay was performed, and mammary gland tissue was visualized at 40×. (**B**,**D**) are representative Ki67-stained mammary gland tissues of CON cows and OMN cows on −21 d relative to calving, respectively. Data are presented as LSM ± SEM (**A**,**C**). * *p* ≤ 0.10.

*Mammary Cell Proliferation.* Epithelial proliferation and total cell proliferation percentage tended to increase when cows received OMN treatment relative to CON (*p* ≤ 0.10, Figure 2). However, no differences were found in the stromal proliferation when comparing CON and OMN. Also, no differences were observed when comparing temperature treatment (HT vs. CL) in the analysis of epithelium, stromal, and total cell proliferation. There was a time effect (*p* ≤ 0.01), where cows had higher epithelial (3.3 ± 0.32% vs. 1.4 ± 0.31%), stromal (4.7 ± 0.41% vs. 1.7 ± 0.49%), and total cell proliferation (4.0 ± 0.36 vs. 1.6 ± 0.35%) on day −21 relative to day −43, respectively (*p* ≤ 0.01).

**Figure 3:**
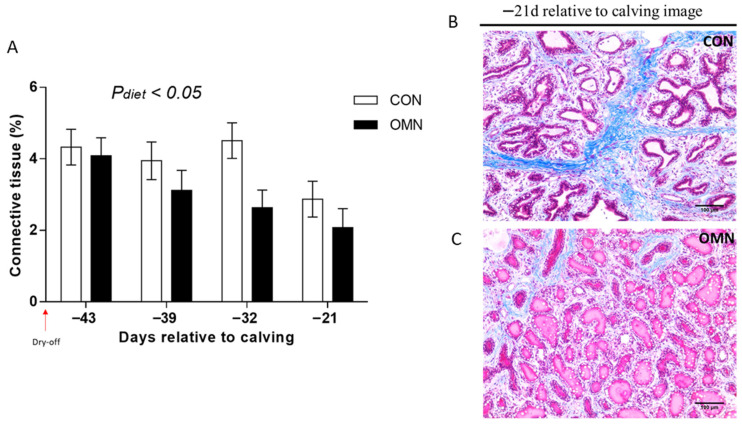
Connective tissue fraction of mammary tissue in Holstein dairy cows supplemented with OmniGen-AF^®^ and exposed to heat stress in the dry period. Multiparous cows were exposed to dietary treatment (CON and OMN, n = 4–6/trt) during the dry period (~46 d before expected calving). Mammary biopsies were collected at days −43, −39, −32, and −21 d relative to calving. For trichrome analysis, Masson’s trichrome-stained mammary gland tissue was visualized at 20×, and the proportions of connective tissue in the mammary gland are represented as LSM ± SEM (**A**). Cows receiving OMN treatment tended to have a lower proportion of connective tissue relative to CON cows ((**A**); *p* ≤ 0.05). (**B**,**C**) represent Masson’s trichrome-stained mammary gland tissue of CON cows and OMN cows on −21 d relative to calving, respectively. The scale bar is 100 μm for Masson’s trichrome staining.

*Trichrome staining.* Trichrome analysis showed the impacts of dietary treatment (CON vs. OMN, *p* ≤ 0.05, Figure 3) and time (*p* ≤ 0.05). Connective tissue was lower on OMN relative to CON, where this effect is more accentuated on day −32 (*p* ≤ 0.01). Also, regardless of temperature or dietary treatment, there was a time effect, and connective tissue was lower on day −21 relative to days −43, −39, and −32. Also, HT + OMN and CL + CON tended to have a higher proportion of connective tissue relative to CL + OMN (*p* ≤ 0.10); however, there was no temperature and dietary treatment interaction (*p* = 0.79).

**Figure 4:**
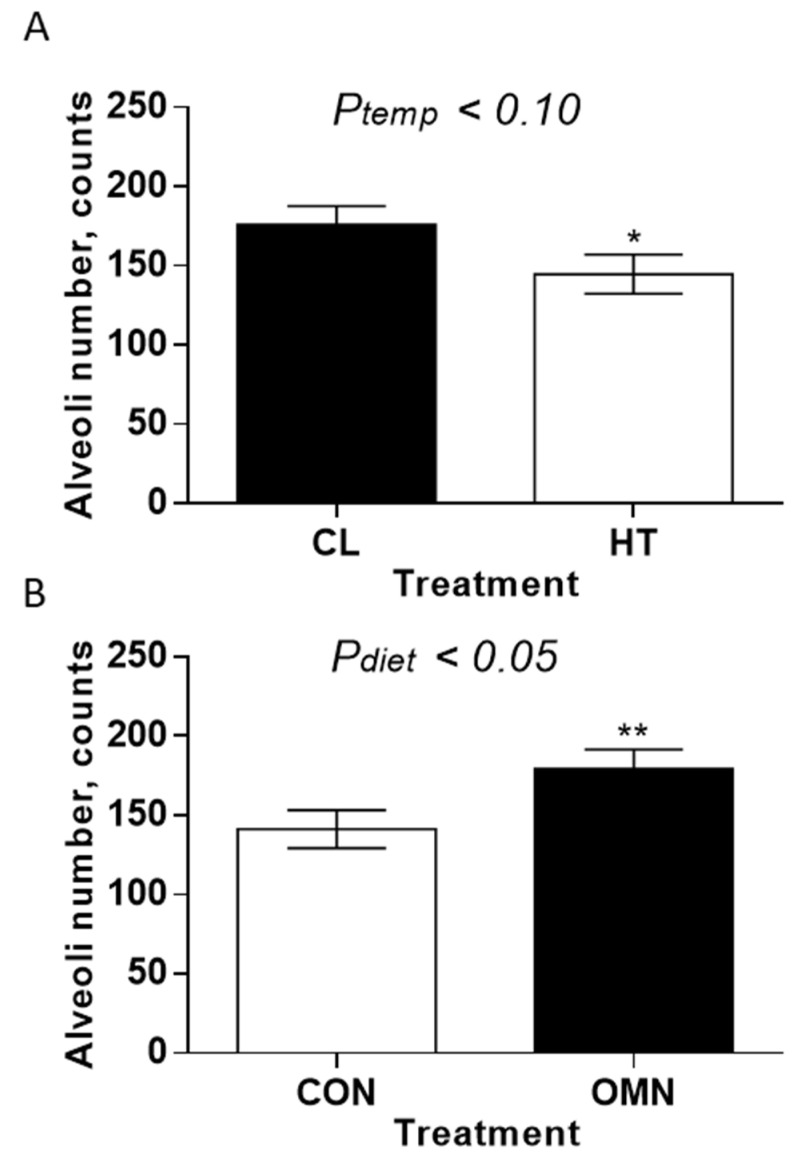
Mammary alveoli number in Holstein dairy cows supplemented with OmniGen-AF^®^ and exposed to heat stress in the dry period. Multiparous cows were exposed to temperature treatment (HT and CL, n = 5–6/trt) or dietary treatment (CON and OMN, n = 4–6/trt) during the dry period (~46 d before expected calving). Mammary biopsies were collected at days −43, −39, −32, and −21 d relative to calving. For alveoli number analysis, hematoxylin and eosin (H&E)-stained mammary gland tissue was visualized at 10×, and alveoli numbers are represented in panels (**A**,**B**) (temperature and dietary treatments, respectively). * *p* ≤ 0.10; ** *p* ≤ 0.05.

*Alveoli number.* The alveoli number was impacted by temperature treatment (HT vs. CL, *p* ≤ 0.10; Figure 4) and dietary treatment (CON vs. OMN, *p* ≤ 0.05; Figure 4). There were more alveoli present in the mammary gland of CL and OMN cows relative to HT and CON cows, respectively. Comparing temperature treatment, this difference was more apparent on day −21 (*p* ≤ 0.01), whereas for the dietary response, the difference was more apparent on days −43, −39, and −32 (*p* ≤ 0.10; *p* ≤ 0.05; and *p* ≤ 0.05, respectively).

### 3.3. Mammary Gland Gene Expression

**Figure 5:**
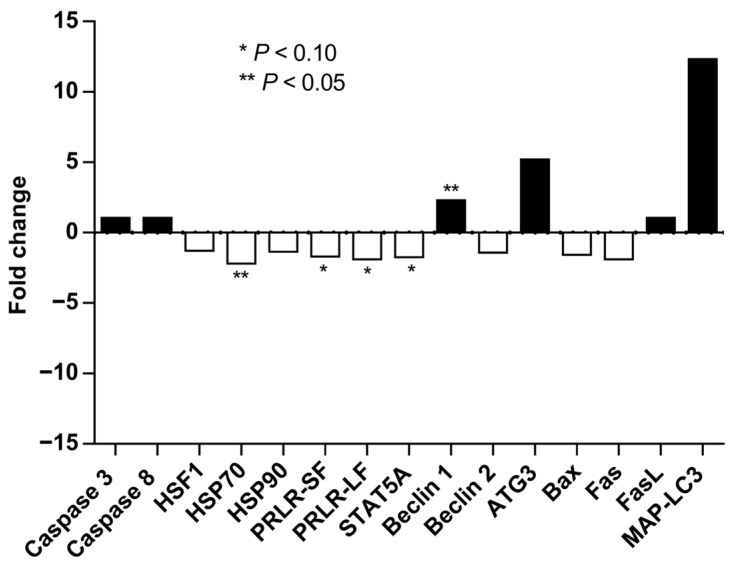
Mammary gland gene expression of Holstein dairy cows supplemented with OmniGen-AF^®^ and exposed to heat stress in the dry period. Multiparous cows were supplemented with dietary treatment (CON and OMN, n = 4–6/trt) for the last 60 days before dry off. Mammary biopsies were collected 3 d before dry off. For gene expression analysis, real-time PCR (qRT-PCR) was performed for the expression of caspase 3 and 8, HSF1, HSP70 and 90, PRLR-SF and LF, STAT5a, BECN1 and 2, ATG3, Bax, Fas, FasL, and MAP-LC3. Fold changes are shown relative to control (CON-treated cows). * *p* ≤ 0.10; ** *p* ≤ 0.05.

*Late Lactation.* In the absence of supplementation, OMN cows tended to have downregulation of *PRLR-SF* and *PRLR-LF*, *STAT5a*, and *HSP70* gene expression relative to CON cows (*p* ≤ 0.10, Figure 5). However, cows that received OMN supplementation upregulated BECN1 gene expression relative to CON cows (*p* ≤ 0.05, Figure 5). There were no differences in the gene expressions of Caspase 3 and 8 (*CASP3* and *CASP8*, respectively), *HSF1*, *HSP90*, *BECN2*, *ATG3*, *Bax*, *Fas*, *FasL*, *MAP-LC3*, *IL8R*, and *CD62L* (*p* ≥ 0.12).

*Dry Period and Lactation.* Compared with CL, HT cows tended to have downregulation of *BECN2* gene expression (*p* ≤ 0.10, Table 3) on day −43, but upregulation of *BECN1* gene expression on day −39. However, on day −39, HT cows tended to have downregulation of *BAX* and *FAS* (Table 3) relative to CL cows. There was no effect of temperature treatment (HT vs. CL) on *HSP70* and *HSP90*, *HSF1*, *CASP3* and *CASP8*, *FasL*, *ATG3*, *MAP-LC3, PRLR-SF* and *PRL-LF*, and *STAT5a* when analyzed. Compared with CON, OMN cows had downregulation of *HSP70* on day −43 (*p* ≤ 0.05, Table 3). Also, on day −39, OMN cows had downregulation of *HSF1*, *BECN2*, *FAS*, *STAT5a,* and *PRLR-SF* (Table 3). There was no effect of dietary treatment (CON vs. OMN) on *HSP90*, *CASP3* and *CASP8*, *BECN1*, *Bax*, *FasL*, *ATG3*, *MAP-LC3*, and *PRLR-LF.* There was no effect of treatment on the expression of *IL8R* and *CD62L* during the dry period and during lactation (*p* ≥ 0.13).

### 3.4. Cortisol and Prolactin Analysis

**Figure 6:**
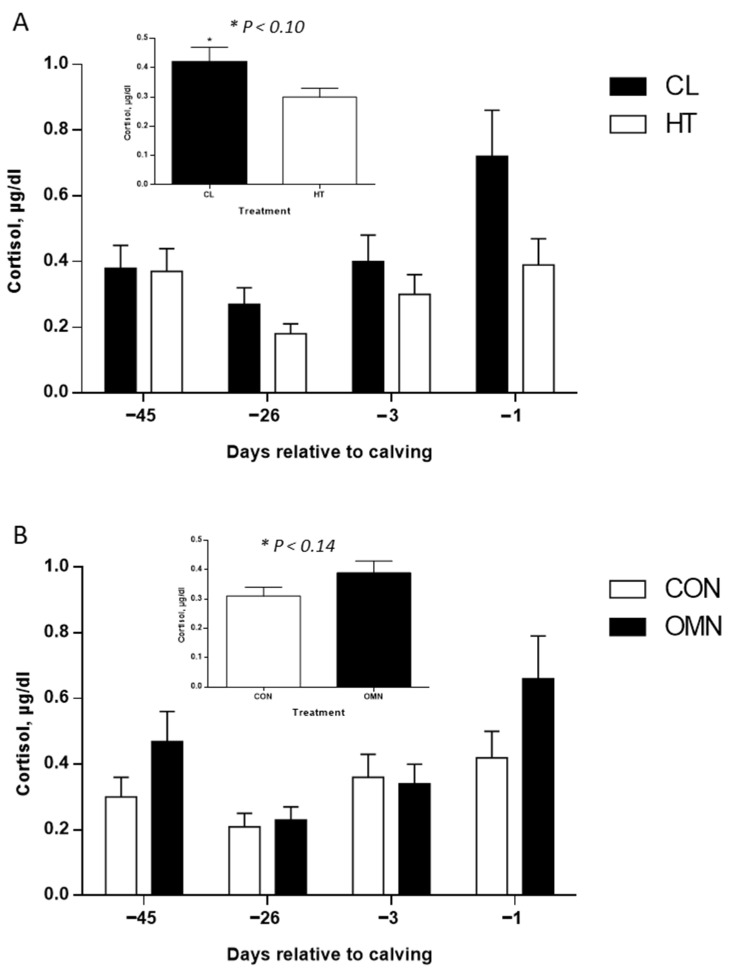
Cortisol concentrations of Holstein dairy cows supplemented with OmniGen-AF^®^ and exposed to heat stress in the dry period. Blood samples were collected from the coccygeal vessels, and samples were used for cortisol concentration analysis. Samples were collected on day 0, when temperature treatments (i.e., CL or HT) were applied (~−46 d relative to calving), on day 1 (~−45 d relative to calving), the mid-dry period (~−26 d relative to calving), and as parturition approached (−3 d and −1 d relative to calving; n = 13 cows per treatment). Samples were analyzed using a chemiluminescent enzyme immunoassay (Immulite 1000; Siemens Medical Solutions Diagnostics, Los Angeles, CA, USA). Insets represent temperature (HT vs. CL, *p* ≤ 0.10) and dietary treatments (CON vs. OMN, *p* ≤ 0.14), and data are represented as LSM and SEM (**A**,**B**).

*Cortisol.* Under HT, cows had lower cortisol concentrations relative to CL (*p* ≤ 0.05, Figure 6). However no differences were found midway through the dry period (~−26 d relative to calving) and 3 days before calving, but there was an inverted response on the day before calving, where HT cows had lower cortisol concentrations relative to CL cows (*p* ≤ 0.01; Figure 6), and higher cortisol concentrations of CL cows seem to be due to the collection 1 d before calving; however, there was no treatment and time interaction (*p* = 0.45). No effect of dietary treatment was found in the analysis (*p* = 0.14). Compared with OMN, CON cows had lower cortisol concentrations, especially one day before calving (*p* ≤ 0.10; Figure 6); however, there were no interactions between dietary and time effects (*p* = 0.40). Also, regardless of treatment, cortisol concentrations spiked one day before calving (*p* ≤ 0.01; Figure 6).

**Figure 7:**
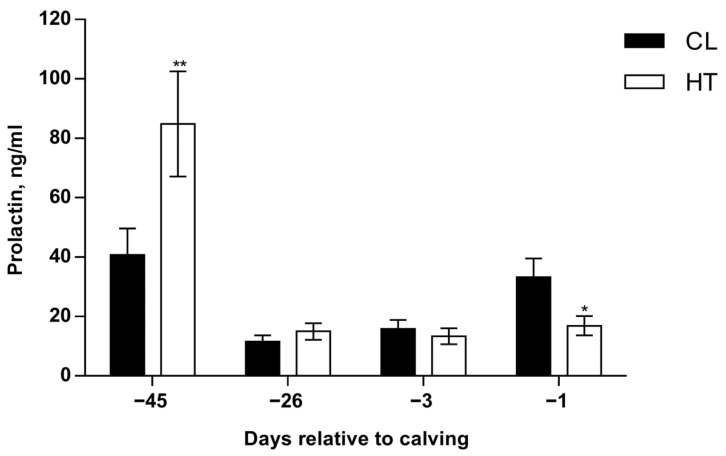
Prolactin concentrations of Holstein dairy cows supplemented with OmniGen-AF^®^ and exposed to heat stress in the dry period. Blood samples were collected from coccygeal vessels, and plasma samples were used for prolactin (PRL) concentration analysis. Samples were collected on day 0, when temperature treatments were applied (~−46 d relative to calving), on day 1 (~−45 d relative to calving), the mid-dry period (~−26 d relative to calving), and as parturition approached (−3 d and −1 d relative to calving). Prolactin was measured by using an Enzyme-Linked Immunosorbent assay kit (Cloud-Clone Corp. catalog number, SEA846Bo) according to the manufacturer’s procedure. * *p* ≤ 0.10; ** *p* ≤ 0.05.

*Prolactin. *There was a temperature and time interaction (*p* ≤ 0.01; Figure 7), where HT cows had a higher PRL concentration 1 day after temperature treatment was applied (*p* ≤ 0.05). However, no differences were found midway through the dry period (~−26 d relative to calving) and 3 days before calving, but there was an inverted response on the day before calving, where HT cows had lower PRL concentrations relative to CL cows (*p* ≤ 0.01; Figure 7). No effect of dietary treatment was observed (*p* = 0.45).

## 4. Discussion

Exposure to heat stress in the dry period results in lower milk yield in the following lactation, a response that is driven by reductions in mammary growth that are associated with immune suppression [3,13]. The cooling of dry cows reverses this yield drag and mammary growth deficit, but whether supplementation with an immunomodulator would alter mammary development when cows experience heat stress is unknown. Heat-stressed dry cows fed OmniGen-AF^®^ produced more milk in the next lactation compared with those fed the vehicle alone (CON; [2]). However, this effect was not apparent when cows were cooled, as there was no difference in the yields of cows on OMN that were cooled during the entire dry period, suggesting that OMN was able to overcome some of the same limitations on mammary growth and development in the dry period that cooling does. In order to fully understand effects at the intersection of hyperthermia, immune status, and mammary development in the dry period, we examined a broad set of immune metrics, mammary gene expression, and indicators of mammary development. In general, the most consistent effects were parallel responses of OMN and cooling relative to the CON diet and heat stress, suggesting some similar pathways for both interventions, but little evidence of any interaction. Therefore, the following discussion is limited to the main treatment impacts of immunomodulator supplementation or heat stress.

During the dry period, dairy cows are highly susceptible to intramammary infections [14,15], and the migration of phagocytic cells early in the dry period may be important to mammary gland involution through clearance of cellular debris. Recent studies have examined the mechanisms involved in mammary involution and redevelopment under heat stress conditions [3,16]. The changes in the mammary gland are not only cellular but also include the intracellular ultrastructure and are consistent with the decrease in milk production by the epithelial cells [6]. However, there is limited information between the connection of mammary gland involution and immune status.

Diez-Fraile et al. [17] previously reviewed the mechanism whereby selectin and integrins are activated in response to a pathogen. Milk resident macrophages respond to a pathogen invasion (i.e., *Escherichia coli*) by releasing inflammatory mediators that lead to a cascade of events that culminate in L-selectin activation. Inflammation is a complex biological mechanism that is triggered in response to a stimulus, i.e., pathogen or tissue injury, which stimulates the recruitment of polymorphonuclear cells to the site of inflammation [18]. During inflammation, L-selectin, an adhesion molecule on the surface of polymorphonuclear cells, plays an important role in rolling and firm adhesion to the endothelial layer and migration of neutrophils to the location of inflammation [19]. However, we believe that this mechanism is still present during involution, even though no inflammatory response occurs. It has been shown that phagocytic activity is higher during mammary gland involution [20], which means that more polymorphonuclear cells are available to migrate to the mammary gland. But it is not clear if more polymorphonuclear cells are migrating to the mammary gland, and it must be further investigated.

We previously observed a broad improvement of immune status with cooling versus heat stress in the dry period [13], and those immune responses are associated with greater mammary cell development with dry period cooling [3]. Cooling and OMN increased mammary cell apoptosis early in the dry period relative to heat stress and CON supplementation, but the cellular fraction affected differed somewhat, with epithelial apoptosis increased in cooled cows and stromal cell apoptosis increased with OMN. When total mammary cell apoptosis was considered, the cooling and OMN treatments both appeared to accelerate cellular loss relative to the heat stress and control dietary treatments.

Mammary connective tissue was greater with OMN vs. CON, suggesting a shift in secretory capacity. We interpret the reduction in connective tissue as an indication of greater relative secretory tissue in the OMN cows relative to CON cows. This is consistent with the idea that more secretory capacity in the mammary gland as cows transition into lactation should support greater yield [3]. A recent study showed that HT during the dry period increases the proportion of mammary gland connective tissue relative to cooled cows during lactation [21]. Also, there is an inverse relationship between connective tissue and alveoli area [22]. Thus, the present study and the literature provide information that supports the idea that heat stress during the dry period alters the dynamics of secretory tissue turnover in the dry period and through lactation and might potentially lead to lower milk yield.

Gene expression patterns with CL and OMN were also partially aligned with the observed effects of each on mammary growth and development in the dry period. For example, downregulation of *BECN2* during involution suggests that HT slows the progression of autophagy relative to CL. A similar pattern of reduced autophagy with HT has been observed previously in this same animal model [16]. Genes related to apoptosis, including *BAX* and *FAS*, were also downregulated in the HT cows relative to CL, which indicates a delay in apoptosis with HT relative to CL.

In contrast, supplementation with OMN resulted in downregulation of *BECN2* in early involution, but that was coupled to lower expression of the protective heat shock proteins associated with cellular survival in response to thermal shock [23]. In the CON cows, greater expression of prolactin signaling-associated genes, including *PRLR-SF* and *STAT5a*, suggests that MEC activity was maintained even as milk removal ended, possibly due to slower cell clearance. Indeed, *FAS* expression was also higher in CON cows during involution, which is further evidence that apoptosis was delayed. Thus, OMN appears to be associated with a greater loss of cells early in the dry period compared with CON due to lower cellular survival signaling.

Cortisol responses observed with OMN and CL are consistent with greater mammary cell differentiation as parturition approaches. Relative to heat stress, cooling increased cortisol, and a similar pattern of elevated cortisol was present with OMN feeding versus CON. Cortisol synergizes with prolactin to induce the second stage of lactogenesis [24], and both OMN feeding and prepartum evaporative cooling improve this capacity in the present study. Regarding prolactin, the results are less clear. As expected, concentrations of prolactin were elevated in the immediate period after transfer to the heating or cooling treatments. Indeed, heat stress is well known to increase prolactin in cattle and other species [3,25]. Although circulating prolactin was not different in the middle of the dry period, there was a separation between HT and CL as parturition approached, and the CL cows had a greater elevation of prolactin at the start of the periparturient prolactin surge. This difference at parturition may reflect a chronic downregulation of prolactin secretion under heat stress in the dry period relative to cooling.

Prolactin not only plays an important role in mammogenesis and lactogenesis in dairy cows [25] but also has an anti-apoptotic effect in mammary cells of mice [26] and cattle [27]. Thus, the increase in PRL with heat stress would be expected to result in lower apoptosis in heat-stressed cows relative to cooled animals. The overall impact of cooling and OMN treatments, however, would be expected to be positively related to the process of lactogenesis and thus mammary cell differentiation.

## 5. Conclusions

In summary, heat stress during the dry period negatively affects the mammary gland turnover of dairy cows, and this study adds additional information that might help explain the mechanism by which heat stress during the dry period reduces milk yield in the next lactation. Following the same trend as in CL treatment, OmniGen-AF^®^ improved mammary gland turnover, which might explain why cows supplemented with OMN have increased milk production, as reported previously [2]. The immune status differences found in this study were somewhat modest, but there are still areas to investigate and understand in the link between mammary gland involution and the immune status of dairy cows, as OMN supplementation has been reported to improve immune status [11,28]. Thus, the relationship among mammary gland involution and remodeling and immune status, and the potential to modulate that relationship with dietary and environmental management, warrants further investigation.

## Figures and Tables

**Table 1:** Primers used in real-time PCR.

Gene Name	Primer	Sequence (5′-3′)	
**GAPDH**	Forward	TGA CCC CTT CAT TGA CCT TC	NM_001034034.2
	Reverse	TAC TCA GCA CCA GCA TCA CC	
**RSP9**	Forward	GGA GAC CCT TCG AGA AGT CC	NW_021639847.1
	Reverse	CTT TCT CAT CCA GCG TCA GC	
**CASP3**	Forward	TGA AAT ACG AAG TCA GGA TTA AA	NM_001077840.1
	Reverse	GTC CGT TGG TTC CAA AAA TG	
**CASP8**	Forward	TTT AGC ATA GCA CGG AAG CA	NM_001045970.2
	Reverse	TAT CCA AAG CGT CTG CAT CA	
**HSF1**	Forward	CAG CTG ATG AAG GGG AAG CA	NM_001076809.1
	Reverse	TGG ATG AGC TTG TTG ACG ACT	
**HSP70**	Forward	GGG GAG GAC TTC GAC AAC AGG	NM_203322.3
	Reverse	CGG AAC AGG TCG GAG CAC AGC	
**HSP90**	Forward	AGG CAG AGG CTG ACA AGA ATG ACA	NM_001079637.1
	Reverse	AGC CAG AAG ACA GGA GAG CTG TTT	
**PRLR-SF**	Forward	AGG TGA CAC TAT AGA ATA AGC AAC	NM_174155.3
	Reverse	GTA CGA CTC ACT ATA GGG AAA GGC	
**PRLR-LF**	Forward	AAG GCC ATG TGG AAG ATT TG	NM_174155.3
	Reverse	GAT GAC TGT GAG GAC CAG CA	
**STAT5a**	Forward	GAA ACA TCA CAA GCC CCA TT	NM_001012673.1
	Reverse	TGA AGC GCA ACA AGA AGG TA	
**BCL1**	Forward	GAT GGA ATA GGA ACC ACC AC	NM_001033627.2
	Reverse	AGT TGA GAA AGG CGA GAC AC	
**BCL2**	Forward	GAG TTC GGA GGG GTC ATG TG	NM_001166486.1
	Reverse	ACA AAG GCG TCC CAG CC	
**BAX**	Forward	CAG GGT GGT TGG GAC GG	NM_173894.1
	Reverse	CTT CCA GAT GGT GAG CGA GG	
**FAS**	Forward	GTT CCC CCA GCT CAA CGA A	AF479289.1
	Reverse	GGA CAT GCT GCT CAA AGG ATG	
**FASL**	Forward	AGT CTG GCC TTT GAC ACC TG	NM_001098859.2
	Reverse	GTC CAC CCA GAA GAT TGG GG	
**MAP-LC3**	Forward	CTG AGG GGA GGC TGC AAA T	XM_005214696.2
	Reverse	GCT AGA TGA CAC AGT GAC G	
**ATG3**	Forward	GGT TGT TCG GCT ATG ATG AG	NM_001075364.1
	Reverse	GGG AGA TGA GGG TGA TTT TC	
**IGFR1**	Forward	GGG CTG AGT TGG TGG ATG	NM_001244612.1
	Reverse	CTC CAG CCT CCT CAG ATC AC	

**Table 2:** Effects of temperature treatment (shade, fans, soakers, CL vs. only shade, HT) and dietary treatment (CON, fed with 56 g/d of placebo during late lactation and dry period vs. OMN, fed with 56 g/d of OmniGen-AF^®^; Phibro Animal Health Corp., Teaneck, NJ, USA) during late lactation and dry period on hematology parameters during the dry period of Holstein dairy cows (n = 6–8 cows/treatment).

Variables	TRT					*p*-Value			
	CL	HT	CL + OMN	HT + OMN	SEM	T1 ^1^	T2 ^2^	DAY ^3^	T1 × T2 ^4^
Red blood cell (×10^6^/µL)	6.2	6.04	6.2	5.8	0.22	0.24	0.6	<0.01	0.58
Hematocrit (%)	31.7	30	31.5	28.7	0.92	0.02	0.41	0.06	0.58
Hemoglobin (g/dL)	5.6	5.1	4.4	4.7	0.34	0.77	<0.01	<0.01	0.11
Platelet count (10^3^/µL)	263.9	264.4	228.1	332.2	27.9	0.06	0.42	0.04	0.07
Reticulocytes (10^3^/µL)	7	5.5	7.8	5.9	2.4	0.48	0.82	0.12	0.96
White blood cell count (×10^3^/µL)	23.7	14.8	20	17.7	6	0.35	0.94	0.71	0.58
Neutrophils (×10^3^/µL)	3.25	3.29	3.81	3.48	0.24	0.55	0.13	<0.01	0.46
Lymphocytes (×10^3^/µL)	9	8	9.3	8.5	3.05	0.78	0.9	0.02	0.98
Eosinophils (×10^3^/µL)	0.71	0.52	0.52	0.78	0.1	0.73	0.74	<0.01	0.04
Monocytes (×10^3^/µL)	1.21	0.95	0.93	0.91	0.21	0.51	0.47	0.02	0.59
Basophils (%)	52.0	30.6	63.1	36.8	15.2	0.14	0.58	0.21	0.90

^1^ T1 = effect of temperature treatments (CL vs. HT). ^2^ T2 = effect of dietary treatment (CON vs. OMN). ^3^ Day relative to calving (−43, −39, −32, and −21). ^4^ T1 × T2 = treatment 1 by treatment 2 interaction.

**Table 3:** Effect temperature treatment (shade, fans, soakers, CL vs. only shade, HT) and dietary supplement treatment (CON, fed with 56 g/d of placebo during late lactation and dry period vs. OMN, fed with 56 g/d of OmniGen-AF^®^ during late lactation and dry period; Phibro Animal Health Corp., Teaneck, NJ, USA) on mammary gland gene expression during the dry period of Holstein dairy cows (3 [d-43] and 7 d [d-39] after dry off, n = 4–6 cows/treatment).

	DAY −43		DAY −43		DAY −39		DAY −39		*p*-VALUE	
GENE *	CL (ddct)	HT (ddct)	OMN (ddct)	CON (ddct)	CL (ddct)	HT (ddct)	OMN (ddct)	CON (ddct)	TEMP	DIET
BECN1	-	-	-	-	3.32	2.11	-	-	<0.10	-
BECN2	5.58	6.11	-	-	-	-	6.52	5.79	<0.10	<0.10
BAX	-	-	-	-	6.31	7.00	-	-	<0.10	-
FAS	-	-	-	-	2.90	3.70	3.84	2.80	<0.10	<0.05
STAT5a	-	-	-	-	-	-	5.05	4.43	-	<0.05
HSP70	-	-	4.28	5.11	-	-	-	-	-	<0.05
HSF1	-	-	-	-	-	-	5.44	5.05	-	<0.01
PRLR-SF	-	-	-	-	-	-	5.61	4.89	-	<0.05

* ddct represents the delta delta CT values.

## Data Availability

The data presented in this study are available on request from the corresponding author.
